# Improving community coverage of Japanese encephalitis vaccination: lessons learned from a mass campaign in Battambang Province, Cambodia

**DOI:** 10.1186/s12889-022-14428-7

**Published:** 2022-12-01

**Authors:** Michael C. Thigpen, Svay Sarath, Sann Chan Soeung, Ork Vichit, Paul Kitsutani, Hardeep Sandhu, Christopher Gregory, Marc Fischer, Chheng Morn, Susan L. Hills

**Affiliations:** 1 Centers for Disease Control and Prevention, Fort Collins, CO USA; 2 Consultant, Phnom Penh, Cambodia; 3 National Immunization Program, Ministry of Health, Phnom Penh, Cambodia; 4 Centers for Disease Control and Prevention, Atlanta, GA USA

**Keywords:** Vaccination, Campaign, Immunization, Cambodia, Japanese encephalitis, Mass campaign, Lessons learned

## Abstract

A mass Japanese encephalitis (JE) immunization campaign for children aged 9 months through 12 years was conducted in 2013 in Battambang province, western Cambodia. Vaccinators working at almost 2,000 immunization posts in approximately 800 villages provided vaccinations to almost 310,000 children using one dose of Chengdu Institute of Biological Products’ live, attenuated SA14-14-2 JE vaccine (CD-JEV), achieving a coverage rate of greater than 90%. Lessons learned, in general for mass vaccination campaigns and specifically for vaccination with CD-JEV, are described. These observations will be of benefit for public health officials and to help inform planning for future campaigns for JE or other vaccine-preventable diseases in Cambodia and elsewhere.

## Background

Japanese encephalitis (JE) is a mosquito-borne neuroinvasive disease. JE is often severe, resulting in death in 20%–30% of patients and disability among 30%–50% of survivors [1]. Vaccination is the only effective long-term control measure for JE [2]. In endemic areas, the World Health Organization (WHO) recommends an initial mass campaign to protect all children in at-risk age groups defined by local epidemiology, followed by incorporation of JE vaccine into the routine immunization program [2]. In 2011, based on the status of vaccination programs at that time, a systematic review estimated that 67,900 JE cases typically occurred annually [3]. Continued progress with JE control is being made through implementation of JE vaccination programs in many Asian countries [4].

JE is endemic in Cambodia, and a JE vaccination program was implemented in Kampong Cham, Svay Rieng, and Takeo provinces in 2009, with vaccine delivered through the routine immunization program to children aged 10–24 months. However, no mass JE vaccination campaign was initially conducted. In 2013, the National Immunization Program of the Cambodian Ministry of Health (MOH) and Battambang Provincial Health Department, with the support of the United States Centers for Disease Control and Prevention, implemented a mass JE immunization campaign in Battambang province for children aged 9 months through 12 years. The campaign was conducted from February 18 through March 14 and included Battambang province’s five Operational Districts (ODs)—Battambang, Sangkae, Moung Russey, Thmar Kol, and Sampov Loun. Staff at almost 2,000 immunization posts in approximately 800 villages provided a single dose of Chengdu Institute of Biological Products’ live, attenuated SA14-14-2 JE vaccine (CD-JEV) to almost 310,000 children. A post-campaign coverage survey, which used a modified form of the WHO-recommended cluster survey methodology, estimated a coverage rate of 91% (*M Thigpen, personal communication*) [5]. Supervision teams met regularly during the campaign to discuss its progress, strengths, and challenges. CD-JEV is increasingly being used in Asia to control JE, and no publication previously has described lessons learned from mass campaigns with this vaccine. We briefly describe the planning and implementation of the campaign and consider key findings, in general and specifically for CD-JEV, to help inform future campaigns for JE and other vaccine-preventable diseases in Cambodia and elsewhere.

## Campaign planning and preparation

### Development of campaign documents

Operational field guidelines were prepared and included information on the vaccine (e.g., administration route, cold chain requirements), strategies for social mobilization, staff and supervisor roles and responsibilities, developing microplans, recording and reporting vaccine administration information, managing waste, and conducting adverse events following immunization (AEFI) surveillance [6]. Other documents developed included campaign vaccination cards, job aids (e.g., “age eligible for vaccination” chart), registration lists, tally sheets, AEFI forms, and supervision checklists.

### Training

Several different trainings were conducted including for Provincial Health Department staff, OD public health staff, health center staff, commune officials, local authorities, and Village Health Support Group (VHSG) members, who are community health workers who provide a connection between the traditional healthcare system and the local community. Training content was based on the information outlined in the operational field guidelines, modified to ensure it appropriateness to each target audience, and relevant training materials were provided.

### Microplanning

Staff in each OD prepared a microplan which outlined the daily schedule for the campaign with information on each village’s target population, number of teams needed, location of the immunization posts, and names of the vaccination team members, VHSG members, and supervisor allocated to each village. The plans included strategies for vaccinating hard-to-reach population groups (e.g., urban poor, residents of remote rural villages, and ethnic minority populations).

### Registration activity

In the weeks prior to the campaign, VHSG members were paid a small allowance to go house-to-house in their villages to prepare a registration list of all children within the eligible age range for vaccination. The lists were intended to provide accurate data on the campaign target population and be used on each village’s vaccination day to mark off children who attended and identify and search for non-attendees.

### Communication and social mobilization

Key local leaders were engaged through direct communication or village meetings. Strategies for social mobilization reflected routine practices, including posters, loudspeaker announcements, and community meetings. In addition, television and radio announcements were prepared that highlighted four specific messages including: 1) JE can be a severe disease; 2) Vaccination is the best way to prevent JE; 3) JE vaccine is safe; and 4) JE vaccine has been used long-term in other countries. Given this was the first mass campaign with CD-JEV in Cambodia, clear messaging was important to avoid the community considering the campaign a type of research clinical trial. A key purpose of developing and disseminating clear and simple messages before the campaign was to reduce the likelihood of vaccine myths emerging, and to enable a timely and informed response by vaccinators if any rumors began circulating during the campaign.

### Supplies

Vaccine supplies were ordered eight months prior to the campaign based on an estimated target population of 300,000 children, expected coverage rate of 95%, and a vaccine wastage rate of 15%. Determining the target population numbers was complicated by variations of almost 20,000 children in estimates from different data sources, and the highest estimate from the MOH’s Health Information System was ultimately used. The vaccine and diluent ordered was in five-dose vials with vaccine vial monitors attached. Auto-disable syringes, safety boxes, and other supplies were also ordered.

## Campaign implementation

### Setting

**Fig. 1:**
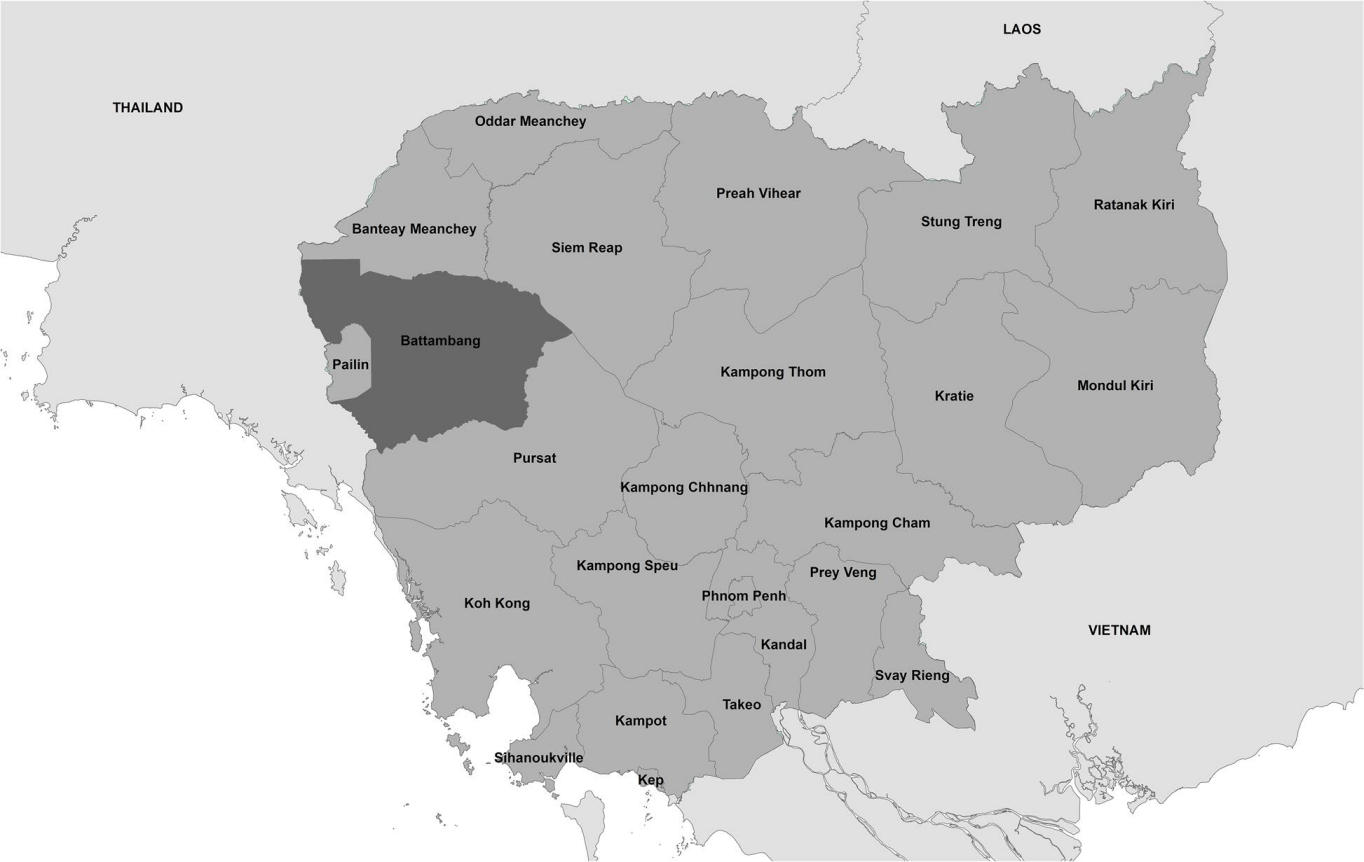
Map showing location of Battambang province in western Cambodia (ArcGIS v10.7.1 [7])

Battambang province is a moderately large province in western Cambodia and the fifth largest in terms of population (Fig. 1). The terrain is varied and includes lowland areas with extensive rice cultivation, mountainous and jungle areas, and some parts of Cambodia’s Tonle Sap Lake where residents live in floating villages.

### Strategy

The campaign commenced in a phased manner, starting on the first day with one OD, on the third day with two additional ODs, and on the fifth day with the remaining two ODs. Campaigns lasted 12–19 days in each OD, depending on its population size, geographical extent, and number of villages. “Mop-up” vaccination activities were also implemented following the campaign, involving visits to homes of children who had not presented for vaccination during the campaign.

### Immunization posts and vaccinator teams

**Fig. 2:**
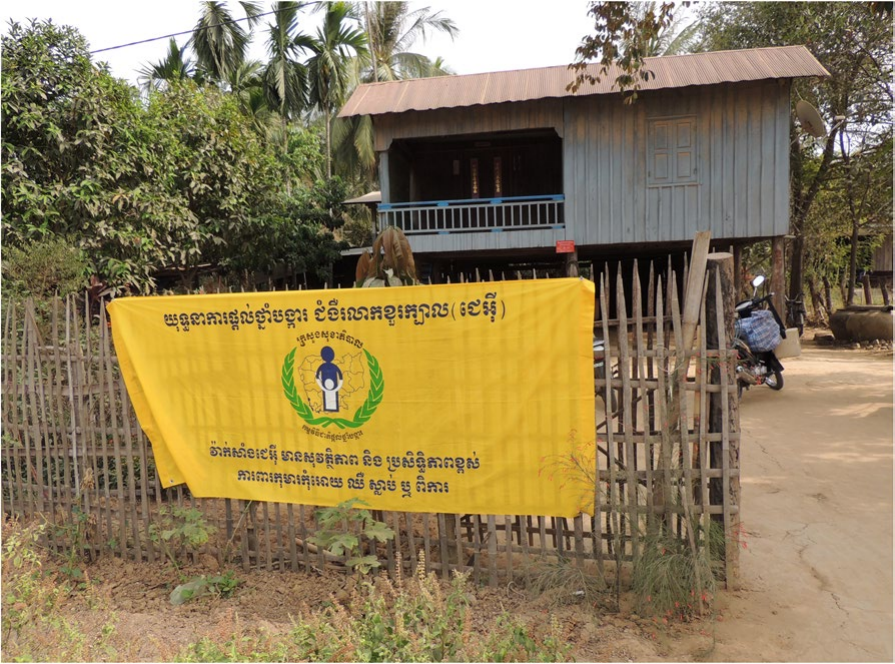
Yellow banner indicating an immunization post for the Japanese encephalitis immunization campaign in Battambang Province, Cambodia

Both fixed immunization posts and mobile vaccination teams were used. In addition to vaccination services at health centers, fixed immunization posts were set up in heavily frequented areas, including at temples, markets, restaurants, houses of villages chiefs or other village members, and in village squares. Each post was marked by a yellow banner and/or poster (Fig. 2). Mobile teams visited the homes of children who could not reach immunization posts. Outreach to facilities with large numbers of children, such as orphanages, was conducted. Immunization activities were also undertaken at most schools.

Each team had two vaccinators with two teams often working concurrently at busy sites. At most posts, a VHSG member was present for some or all of the day; their role varied by site but often included checking off children on the register, assisting with completion of immunization cards, site management, or visiting homes of non-attendees to encourage vaccination. At some sites, an additional community member such as the village chief was also present. Vaccinators, VHSG members, and supervisors wore yellow campaign T-shirts to enable them to be easily recognized.

### Vaccine preparation and administration

A vaccinator prepared the five-dose vaccine vial by mixing the 2.5 mL of diluent with the lyophilized vaccine. On each reconstituted vaccine vial, the vaccinator wrote the time it should be discarded based on the open multi-dose vial policy at that time (i.e., discarded 2 h after opening). There was some variability from site to site, but one vaccination team member typically administered vaccine, drawing up each dose immediately prior to administration and then injecting the dose subcutaneously into a child’s upper arm. After vaccination, the second vaccinator marked a tally sheet to record the vaccination, used gentian violet dye to mark the child’s index finger, and completed the child’s immunization card; this was either the routine card, if available, or a specific campaign card. For routine vaccination activities in Cambodia, documentation of consent is not required. Ethics approval was not required as the immunization campaign was not a study or research activity.

### Logistics

Vaccine was stored in chest refrigerators at the Provincial Health Department and in cold boxes with ice at each OD. Diluent was stored in cardboard boxes and typically chilled for about 24 h before use. Each morning, vaccination plans were reviewed by teams and supplies were distributed as needed from the OD or a health center distribution point. Vaccinators usually carried supplies to the immunization posts on their motorbikes; for some locations, transportation by four-wheel-drive vehicle or boat was required. Remaining supplies, used safety boxes, tally sheets, and reporting forms were returned to the central distribution point each afternoon.

### Monitoring and supervision

Primary supervision was provided by Provincial Health Department and OD staff, with each supervisor being responsible for approximately four vaccination teams. The supervisors visited teams and used a standard supervision checklist to monitor progress, identify any problems, and suggest corrective action. One three-person senior supervision team, consisting of a National Immunization Program staff person and two local or international technical advisors, was assigned to each OD and visited immunization posts each morning. Primary and senior supervision teams also conducted village rapid coverage assessments. For these assessments, survey methodology followed an approach similar to the Expanded Program on Immunization-style “random walk” method [8]. The team members started at the village chief’s house or another house chosen in consultation with a senior village member (avoiding those close to the immunization post), spun a pen and noted the direction it was pointing, counted the number of houses based on a pre-selected random number, and began the survey at that house. The team then went to the nearest house in the same direction and continued until the vaccination status of at least 20 children had been checked based on vaccination cards or gentian violet on children’s fingers.

## General observations and lessons learned

Overall, good management and monitoring by Provincial Health Department staff, hard work of vaccination teams, and the commitment of local authorities facilitated a campaign that achieved high vaccination coverage. The frequently large crowds at immunization posts demonstrated good population awareness of JE and high demand for vaccination. Key lessons learned are described below and summarized in Table 1.

**Table 1:** Summary of key lessons learned from a mass Japanese encephalitis (JE) vaccination program in Battambang province, Cambodia

LESSONS LEARNED	COMMENT
**General campaign-related**	
1. Pre-prepared registration lists had benefits and challenges	Development of registration lists raised community awareness of the campaign and sometimes allowed follow-up of unvaccinated children; however, they were less effective than anticipated and costs were significant
2. Active support from local authorities was important	Vaccine demand appeared lower when village chiefs provided only passive support to the campaign
3. Phased campaign implementation allowed early problems to be addressed	With this strategy, initial close supervision and problem-solving in individual locations could be provided
4. Numerous easily identifiable posts in a variety of settings made vaccination accessible	Many immunization posts in locations convenient for parents likely contributed to high vaccination coverage
5. Well-organized posts provided safer and more effective vaccination services	Including practical guidance as part of training sessions might improve post management
6. Explaining the importance of careful reporting might improve accuracy	Records are more likely to be a true indicator of a child’s, and the community’s, vaccination status if they are completed after, rather than while awaiting, vaccination
7. Supervisors must have the authority to independently make decisions	Select supervisors with management skills if possible, provide decision-making authority, and cover common management scenarios in training
8. Good adverse event following immunization (AEFI) management is necessary to mitigate any negative impacts on the campaign	AEFI should be expected during mass campaigns from incidental or possibly related medical events, and resources for prompt investigations and management must be available
9. Comprehensive plans are required for hard-to-reach groups	Microplans need to be detailed and outline the financial and staff resources needed to facilitate implementation
**JE vaccine (CD-JEV)-specific**	
1. A practical demonstration of subcutaneous vaccine administration technique would be beneficial	Do not assume experienced vaccinators have strong technique
2. Vaccinators should be instructed on complete diluent withdrawal and introduction into the vaccine vial	Wastage rates and vaccine supplies can be impacted if fewer vaccine doses are available per vial
3. Clear messaging is needed for awareness that it is beneficial for children previously vaccinated with mouse brain-derived JE vaccine to receive CD-JEV	Vaccinators might not be aware that CD-JEV can safely be given to person previously JE-vaccinated and is valuable to boost immunity
4. Campaigns should immediately be followed with incorporation of JE vaccine into the routine immunization campaign	JE vaccine incorporation into the routine program will allow vaccination of children missed during the campaign and sustain immunity among risk groups to avoid the need for additional future campaigns

*JE* Japanese encephalitis, *CD-JEV* Live attenuated SA14-14-2 JE vaccine manufactured by Chengdu Institute of Biologic Products

### Benefits and challenges were apparent with using registration lists prepared in advance of the campaign

The lists that were intended to be a useful tool on vaccination day and to provide accurate information on campaign target population were not as effective as anticipated for several reasons including: 1) During a busy immunization session, there was often inadequate time for staff to locate each child on a paper list with hundreds of names; 2) In many villages there was more than one immunization post, but only one available registration list as the list had not been photocopied or could not be copied as no photocopier was accessible; 3) Children did not always receive vaccination in the village where their house was located; 4) School-aged children generally received vaccination at school posts but registers were usually held at the village posts; and 5) If a different VHSG member than the one who prepared the list attended on campaign day, they were sometimes unfamiliar with how the list had been organized, resulting in difficulties locating children’s names on the list.

Parents frequently reported becoming aware of the campaign through a VHSG member, so the house-to-house visits for the registration activity clearly motivated parents to have their children immunized. However, registration of each child was time consuming for VHSG members and the training and implementation of the activity were costly. Finally, not all VHSG members prepared lists or lists were incomplete, so they could not be used to provide a precise population denominator.

Electronic searchable lists would have addressed many of the problems described but were not feasible at the time. In general, the process of development of the registration lists provided an excellent opportunity for awareness-raising for the campaign and were sometimes helpful in allowing identification and follow-up of unvaccinated children. However, these benefits could have been realized using other community awareness strategies without the significant costs of the registration activity.

### Active support from local authorities was important

In general, local authorities were strongly committed to the campaign. Some village chiefs provided passive support while others were actively engaged, organizing transport for children for vaccination or repeatedly making announcements by loudspeaker when the vaccination team was present. Subjectively, the demand for vaccination was higher when village chiefs were actively engaged in the campaign.

### Phased implementation allowed initial problems to be well-managed

The staggered campaign start allowed close supervision of initial activities in each of the five ODs and the opportunity to implement corrective measures quickly as issues arose. For example, individual vaccinator teams initially made decisions on vaccination for children who were not permanent residents of the province (e.g., children of itinerant farm workers). When the issue was identified, the National Immunization Program staff developed a policy to ensure a consistent approach across ODs. Similarly, misunderstandings regarding the upper age limit for vaccination eligibility were identified at an early stage. The “eligible age” chart was modified to incorporate the birth year animal (e.g., “year of the dragon”) given the greater familiarity of parents with these zodiac signs than with the birth year.

### Large numbers of easily identifiable posts in a variety of settings made vaccination accessible

Accessibility was good because immunization posts were in locations that were convenient for parents such as at markets and temples. The posts were also readily identifiable due to the highly visible yellow banners.

### Well-organized posts provide safer and more effective vaccination services

The quality of immunization post management was variable. If the vaccination team and VHSG member ensured an ordered process of registration and vaccination at the post, vaccination was conducted safely, errors were minimized, and children were vaccinated in a timely way, even at busy sites. Conversely, mistakes were observed at poorly organized sites, including vaccinators forgetting to complete or return immunization cards, failing to mark the child’s finger with gentian violet to indicate completed vaccination, or placing safety boxes in inconvenient locations increasing the risk of needle stick injuries to themselves or others. Poorly managed posts were often observed to be left unattended at lunch for long periods, requiring families to wait for extended periods; basic signage indicating when the vaccinator team would return would have avoided frustration for parents and children. It would be beneficial to included clear guidance on immunization post management, ideally by practical demonstration, in training sessions.

### Accuracy of reporting and reporting might be improved by emphasizing and explaining their importance

Several errors were noted in recording and reporting including tally sheets recording more children vaccinated than possible based on the number of vaccine vials used, and unvaccinated children departing with a completed immunization card before they had received vaccine. These errors typically occurred because the tally sheet or card was prefilled at the time the parent and child arrived, but they departed before the child was vaccinated because of long delays at busy posts. In addition, vaccination teams might have viewed tally sheets as an administrative requirement rather than an evaluation tool that could be used to identify subpopulations with lower-than-expected vaccination coverage needing additional follow up efforts. Training sessions could include explanations to vaccinators on the reasons for and importance of accurate recording and outline best practices. Completing requisite records after vaccination could be encouraged, ideally accomplished by a dedicated staff person. Electronic registers could be a useful tool to reduce the need for handwritten documents; secure, free, data collection tools that can be used offline are now readily available [9–11].

### Ensuring supervisors have the authority to independently make decisions will allow greatest efficiency of campaign implementation

Primary level supervisors needed to actively manage vaccinator teams and resources during the campaign including reassigning teams to different posts to avoid overwhelm or underutilization of various teams, moving posts to more convenient physical locations, rearranging poorly organized sites, and redistributing supplies between sites. Some primary supervisors were uncomfortable with these roles, likely because the role was at a higher authority level than their routine job. It would be desirable to select staff with management skills for supervisor roles and empower them to make decisions. Training could include discussion of common scenarios that might arise during a campaign and practice with managing them.

### A timely and competent response to AEFIs is necessary and having dedicated staff to manage AEFI reports might be helpful

AEFI reports should be expected during mass immunization campaigns when incidental or possibly related medical events will occur following vaccination, simply based on the vast number of people being vaccinated. Rumors about AEFIs following vaccination can rapidly spread, and can result in community loss of confidence in the vaccine. Investigating AEFIs promptly, managing them competently, and disseminating accurate information, can reduce the chance of any impact on vaccine acceptance [6]. Busy vaccination teams might not have capacity to respond immediately to a reported AEFI; therefore, it would be optimal to have at least one trained investigation team assigned to manage AEFI surveillance and respond to reported AEFI to mitigate any negative impacts on the campaign.

### Comprehensive plans are required for hard-to-reach groups

Microplans developed prior to the campaign identified hard-to-reach groups in the province. However, specific plans outlining necessary personnel time, travel requirements, and costs to vaccinate these groups were not prepared, potentially impacting vaccination rates. Including detailed plans as a standard component of district campaign microplans will help to ensure adequate financial and staff resources are allocated. Alternatively, distinct outreach efforts separate from the routine campaign activities could be considered.

## Specific observations and lessons learned related to vaccination with CD-JEV JE vaccine

### Give a practical demonstration showing the correct subcutaneous vaccine administration technique to all vaccinators

**Fig. 3:**
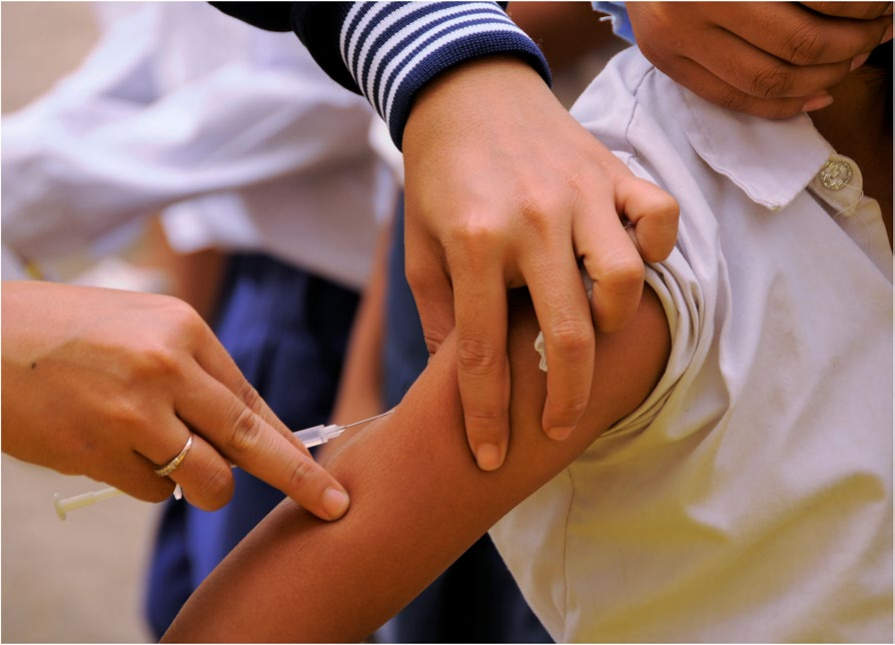
Live, attenuated SA14-14-2 Japanese encephalitis vaccine being injected with the correct subcutaneous technique during the vaccination campaign

CD-JEV is administered subcutaneously, requiring an injection at a 45 degree angle into subcutaneous tissue (Fig. 3). Most vaccinators were staff who gave routine injections at health centers, so training sessions did not always include practical instruction on vaccination technique as competency was assumed. However, intramuscular injections are more commonly given by healthcare staff as part of routine immunization activities, and incorrect administration procedures were occasionally observed during the campaign, even among experienced vaccinators. Training session can be used to give a practical demonstration of subcutaneous injection technique and the appropriate administration site.

### Instruct vaccinators to ensure complete diluent withdrawal and introduction into the vaccine vial

CD-JEV vaccine and diluent were supplied in five-dose vials, and if vaccinators did not take care to transfer the full 2.5 mL of diluent into the vaccine vial, only four vaccines dose were available for administration. If this happens frequently, it can impact vaccine wastage rates and supplies.

### Clearly convey information that it is safe and beneficial for children previously vaccinated with mouse brain-derived JE vaccine to receive CD-JEV

Although JE vaccine previously had limited availability in Cambodia, some children had received a complete or incomplete series of mouse brain-derived JE vaccine prior to the campaign. This vaccine requires two primary doses and boosters to ensure long-term protection. Children who had previously received JE vaccine were encouraged to receive CD-JEV during the campaign, as it can substantially boost JE virus neutralizing antibody levels and is safe [12]. However, during rapid assessments of vaccination coverage in villages, there was evidence that previously vaccinated children were less likely to have received CD-JEV. Making clear the safety and value of vaccination with CD-JEV for previously JE-vaccinated children will likely improve vaccination coverage in these children.

### Immediately follow campaigns with incorporation of JE vaccine into the routine immunization program

WHO notes the most effective JE immunization strategy is a one-time campaign in the primary target population followed by incorporation of JE vaccine into the routine childhood immunization program [2]. The primary reason a child did not receive vaccine during the campaign was that the family was away (*M Thigpen, personal communication*). Although “mop-up” vaccination activities were conducted, some children remained unvaccinated as their families were long-term absentees. Incorporation of JE vaccine into the routine immunization program immediately following a campaign will provide an opportunity to vaccinate children missed during the campaign as well as sustain immunity in newly at-risk children without necessitating additional campaigns.

## Conclusions

Despite a generally well-managed, successful campaign that achieved high coverage, several important lessons were learned during the campaign. These lessons will be beneficial as Cambodia and other countries plan and implement future mass campaigns to prevent JE and other vaccine-preventable diseases. There has been a substantial increase in use of JE vaccine during the last decade, aided by prequalification of CD-JEV and Gavi Alliance funding [13, 14]. Mass campaigns can be resource-intensive, time-consuming and challenging, but there are substantial benefits of rapidly protecting the children at highest risk of infection. The campaigns need to be followed by incorporation of the vaccine into the childhood immunization program in order to sustain protection in at-risk populations. The limitation of these results is that they are based on the observations and experiences of campaign supervisors. Nonetheless, the findings provide helpful information and practical considerations for public health officials planning similar mass campaigns.

## Data Availability

Data sharing is not applicable to this article as no datasets were generated or analysed during the current study.

## Abbreviations

AEFI: Adverse events following immunization
CD-JEV: Chengdu Institute of Biological Products’ live, attenuated SA14-14-2 JE vaccine
JE: Japanese encephalitis
MOH: Ministry of Health
OD: Operational District
VHSG: Village Health Support Group
WHO: World Health Organization
